# Toward Public Health Wellness: Psychosocial & Physical Health in the Community

**DOI:** 10.3390/ijerph19095188

**Published:** 2022-04-25

**Authors:** Won Ju Hwang, Mi Jeong Kim

**Affiliations:** 1 College of Nursing Science, Kyung Hee University, Seoul 02447, Korea; hwangwj@khu.ac.kr; 2 School of Architecture, Hanyang University, Seoul 04763, Korea

## 1. Introduction

Public health plays a pivotal role in society. It is not only the primary source of wellness for most people but is also an essential factor in shaping the quality of life and social identity. Public health disability represents a serious public health problem in all developing and industrialized countries and an enormous economic burden on society [1]. Psychosocial and physical health disorders are the leading causes of wellness problems, with quality of life connected to occupational, social, and economic factors in the community [2]. Public health in the community can meet the specific needs of community members and may even improve and/or reduce significant mental and physical health problems.

This issue focuses on how wellness can be achieved in terms of the public health environment, community care in the workplace, and information technology. Psychological health is often used interchangeably with mental health care and is mainly considered for stress management. In addition, this special issue considers smart care for public wellness, since nursing and public health are integrated with information technologies to support public health and such integration can support public health promotion. Information technology can improve overall quality of life by promoting workers’ wellness and satisfaction. This special issue includes research contributions on wellness care in public and community health. The purpose is to highlight issues of wellness in public health and community care by emphasizing psychological and physical health promotion perspectives.

Fourteen manuscripts have been published on different topics related to psychological and physical health in the community (eight papers) and public health wellness (six papers). Five papers provide innovative findings in relation to psychological health and review composition features [3,4,5,6,7], three papers focus on physical health performance aspects [8,9,10], and six examine factors influencing wellness in different communities, such as employees, older adults, patients, refugees, and the public [11,12,13,14,15,16].

## 2. Materials and Methods

First, a systematic review aimed to examine the effects and results of mental health mobile apps on the general adult population [3]. These apps cover breathing training, meditation, and music therapy. Stress, depression, and anxiety was found to decrease when using these apps, and some were effective in improving well-being. Many mobile apps have been developed with the rapid development of technology related to mental health, but apps based on theoretical knowledge and well-designed research are lacking [17]. Further research and practice should be conducted to develop, test, and disseminate evidence-based mHealth for mental health promotion.

This study provided a systematic review of the efficacy of heart rate variability (HRV) biofeedback in the treatment of mental health disorders such as anxiety, depression, and stress-related disorders [6]. It found a significant improvement in non-invasive HRV biofeedback training in stress-related disorders, such as depression and panic disorder, particularly when combined with cognitive behavioral therapy. The effects were visible after four weeks of training, but clinical practice with a longer daily self-treatment of eight weeks is more promising.

Although health applications have been developed and reviewed in many countries, no baseline study has been carried out on the percentage of Korean workers using these types of health apps. This study investigated work-related stress, health status, and the utilization of health applications among adult Korean workers. Only about 33.7% of the sample used mHealth applications [4]. Therefore, utilization of health applications as health and stress management tools should be encouraged to promote public health.

However, from the perspective of physical health, some community-based studies have evaluated the relationship between insulin resistance (IR) and cardiovascular disease (CVD) risk [8] and between metabolic syndrome and related factors [9] among the Taiwanese population. A previous study found that IR is an independent factor, but it cannot be used solely for evaluating CVD risk. Further prospective cohort studies are warranted to better assess the relationship between CVD risk and IR. A later study found that the factors significantly associated with metabolic syndrome were older age, lower education level, and high levels of uric acid, alanine transaminase (ALT), gamma-glutamyl transferase (γ-GT), and creatinine. A higher prevalence of metabolic syndrome and substance use were observed in the indigenous population than in the urban Taiwanese population (and especially in women).

Another community-based study in Taiwan [10] investigated the association between high-sensitivity C-reactive protein (hs-CRP) and renal impairment among middle-aged and elderly people. The prevalence and odds ratio of renal impairment significantly increased across the hs-CRP tertiles from low to high, and this trend remained significant after adjusting for conventional cardiometabolic risk factors. These findings suggest that renal function should be carefully evaluated in at-risk individuals with elevated hs-CRP levels.

A study in the USA explored the association between food insecurity and depression among early care and education workers, a vulnerable population often working in precarious conditions [7]. Low food security was associated with a higher probability of depression. Policies and center-level interventions that address both food insecurity and depression may be warranted to protect and improve the health of this vulnerable segment of the US workforce.

Another vulnerable population group is international students. During the coronavirus disease (COVID-19) pandemic, governments have updated quarantine guidelines for infection control. However, some international students had difficulty understanding the official announcements made by the country’s government. This study investigated the preventive practices of international students during quarantine and found that attitudes and trust in the quarantine system could affect personal preventive practices during the outbreak of a highly contagious disease [5].

Employees’ perceived accessibility of maternity and childcare leave is a crucial factor influencing community wellness. This study empirically examines why some women are more likely than others to perceive policies as inaccessible and why the accessibility of leave policies in Korea is significantly correlated with women’s employment status and wage level in the labor market [11].

The use of interactive technologies can improve the wellness of the senior community. However, adoption by the elderly in their lives is a significant consideration. This study identified the key factors influencing initial engagement with interactive technology for older adults through focus group discussions, in-depth interviews, observations, and diary studies [12].

Patient clothing can significantly influence patient wellness in the hospital environment. This study investigated whether a pattern using hospital identity elements can affect patients’ emotional responses and contribute to healing [13]. The results show that patient clothing can be a medium which enables patients to positively perceive the quality of medical services.

Temporary housing in disaster areas serves as basic shelter for refugees, wherein they can recover from their suffering. Therefore, securing habitability in shelter planning is the most critical aspect to consider for a community’s wellness. This field study was conducted with victims, staff, and volunteers to identify problems and needs regarding shelter space planning [14].

As climate change accelerates, the global community has encouraged sustainable resilience in our environment by connecting the community with natural resources and biodiversity. This study emphasized the potential of biophilic design for sustainable and resilient residential regeneration and clarified the applicable features of biophilic design in various fields, such as architectural planning, technology, and service, in terms of a residential regeneration scale [15].

Cardiorespiratory fitness (CRF) evaluation is essential to detect vital signs associated with several diseases. Thus, many health applications enabling CRF have been developed to support public wellness. This study examined the reliability and reproducibility of a self-administered 6-min walk test (6 MWT) in asymptomatic adults, using a free smartphone app for step counting since the 6 MWT is a simple, inexpensive, and safe test performed according to international standardization [16].

## 3. Conclusions

To conclude, in addition to growth and development affecting physical and psychological characteristics, food insecurity, cardiorespiratory fitness, and environmental factors such as clothing and housing influenced public health and wellness in the community. This special issue reveals that the evolution of psychological and physical health as well as wellness in public health has a multifaceted nature and that several evaluation and intervention strategies, such as information technology, are currently available to researchers. Further research and practice should be conducted to develop, test, and disseminate evidence-based interventions for public health promotion. Randomized controlled and integrated studies are needed to expand the application of psychological and physical health in various populations in the community toward public health wellness.
